# Effect of adalimumab on neutrophil function in patients with rheumatoid arthritis

**DOI:** 10.1186/ar1477

**Published:** 2005-01-10

**Authors:** Franco Capsoni, Piercarlo Sarzi-Puttini, Fabiola Atzeni, Francesca Minonzio, Paola Bonara, Andrea Doria, Mario Carrabba

**Affiliations:** 1 Department of Internal Medicine, Ospedale Maggiore Policlinico, IRCCS, University of Milan, Milan, Italy; 2 Rheumatology Unit, Ospedale L Sacco, University of Milan, Milan, Italy; 3 Division of Rheumatology, University of Padua, Italy

**Keywords:** adalimumab, neutrophils, rheumatoid arthritis

## Abstract

Neutrophils are known to be targets for the biological activity of tumour necrosis factor (TNF)-α in the pathogenensis of rheumatoid arthritis (RA). Therefore, these cells may be among the targets of anti-TNF-α therapy. In this study we evaluated the effect of therapy with adalimumab (a fully human anti-TNF-α mAb; dosage: 40 mg subcutaneously every other week) on certain phenotypic and functional aspects of neutrophils obtained from 10 selected patients with RA and 20 healthy control individuals. Peripheral blood neutrophils were obtained at baseline and during anti-TNF-α therapy (2, 6 and 12 weeks after the first administration of adalimumab). All patients had been receiving a stable regimen of hydroxychloroquine, methotrexate and prednisone for at least 3 months before and during the study. Baseline neutrophil chemotaxis was significantly decreased in RA patients when compared with control individuals (*P *< 0.001). Two weeks after the first administration of adalimumab, chemotactic activity was completely restored, with no differences noted between patients and control individuals; these normal values were confirmed 6 and 12 weeks after the start of anti-TNF-α therapy. Phagocytic activity and CD11b membrane expression on neutrophils were similar between RA patients and control individuals; no modifications were observed during TNF-α neutralization. The production of reactive oxygen species, both in resting and PMA (phorbol 12-myristate 13-acetate)-stimulated cells, was significantly higher in RA patients at baseline (*P *< 0.05) and was unmodified by anti-TNF-α mAb. Finally, we showed that the activation antigen CD69, which was absent on control neutrophils, was significantly expressed on neutrophils from RA patients at baseline (*P *< 0.001, versus control individuals); however, the molecule was barely detectable on cells obtained from RA patients during adalimumab therapy. Because CD69 potentially plays a role in the pathogenesis of arthritis, our findings suggest that neutrophils are among the targets of anti-TNF-α activity in RA and may provide an insight into a new and interesting mechanism of action of anti-TNF-α mAbs in the control of inflammatory arthritis.

## Introduction

Tumour necrosis factor (TNF)-α has been found to play a central role in the pathogenesis of rheumatoid arthritis (RA), which has led to the rational development of novel drug therapies that neutralize the deleterious effects of this cytokine [1,2]. Several studies have shown dramatic therapeutic effects of anti-TNF-α antibodies, both in experimental collagen-induced arthritis and in the treatment of inflammatory diseases such as rheumatoid arthritis (RA) [3-5], psoriatic arthritis [6], juvenile rheumatoid arthritis [7] and Crohn's disease [8]. The role played by phagocytic cells in the pathogenesis of these inflammatory diseases [9-11] and the capacity of TNF-α to prime and/or activate phagocytic cells [12] suggest, at least in part, that downregulation of phagocyte activity may be involved in the mechanism of action of anti-TNF-α therapy [9].

There is increasing evidence that inhibition of TNF-α may be associated with the development of adverse consequences such as carcinogenesis, autoimmune disorders and, importantly, infectious diseases caused by Gram-positive and Gram-negative bacteria, mycobacteria and fungi (for review, see Olsen and Stein [2]). Again, the role played by TNF-α in the activation of phagocytic cells and the involvement of these cells in the host defence against infections suggest that impairment in phagocytic cell activity may heighten the risk for infection during TNF-α neutralization [13].

Few data have been reported on the effect of anti-TNF-α therapy on neutrophil function *ex vivo*. Decreased influx of neutrophils in inflamed joints was reported by Taylor and coworkers [14] in RA patients treated with infliximab (a chimeric anti-TNF-α mAb) and by Den Broeder and coworkers [15] in patients treated with adalimumab (a fully human anti-TNF-α mAb). However, no significant impairment in *ex vivo *neutrophil function was observed in RA patients treated with etanercept (a soluble human p75 TNF receptor) [16] or with adalimumab [15].

In this work we evaluated certain phenotypic and functional aspects of neutrophils obtained from RA patients during treatment with adalimumab. To this end, chemotaxis, phagocytosis and reactive oxygen species (ROS) production were assessed in peripheral blood neutrophils, together with membrane expression of CD11b and CD69 – two functionally different activation molecules [17].

## Methods

### Reagents

The anti-CD69 mAb (IgG_2a_, clone HP-4B3) was obtained from Calbiochem (La Jolla, CA, USA). The anti-CD11b was OKM1 (mouse IgG_2 _isotype; Ortho Diagnostics, Raritan, NJ, USA). FITC-conjugated goat anti-mouse IgG was from Immunotech SA (Marseille, France). Irrelevant class-matched mAbs were used as controls for nonspecific binding (Becton Dickinson, San Jose, CA, USA). Lymphoprep gradient (density 1.077 g/ml) was purchased from Nyegaard (Oslo, Norway). RPMI 1640 was obtained from HyClone Laboratories (Logan, UT, USA). Bovine serum albumin (BSA), *N*-formyl-methionyl-leucyl-phenylalanine (FMLP), phorbol 12-myristate 13-acetate (PMA), lucigenin (bis-*N*-methylacridinum nitrate) and zymosan A were from Sigma Chemical Company (St. Louis, MO, USA).

### Patients

Peripheral blood samples were collected from10 selected and consenting RA patients who satisfied the American College of Rheumatology 1987 criteria [18], who had active disease (defined as a disease activity score 28 > 3.2) [19], and who were enrolled in a European open-label, multicentre, multinational phase IIIb study (the Adalimumab Research in Active RA [ReAct] study [20]). The study was approved by the ethical committee of the Ospedale L Sacco (Milan, Italy). The mean age of the patients was 61.4 years (range 40–83 years); eight were rheumatoid factor positive and two were rheumatoid factor negative. Three months before and during the study, all patients received hydroxychloroquine (200 mg twice daily), methotrexate intramuscularly (7.5–15 mg/week), and no more than 10 mg/day prednisone. Adalimumab was administered subcutaneously every other week (40 mg). Peripheral blood samples were obtained before anti-TNF-α therapy and immediately before administration of adalimumab at weeks 2, 6 and 12. Controls were 20 healthy individual who were matched to the patients with respect to age and sex.

### *Ex vivo *neutrophil function

Peripheral blood neutrophils were obtained by density gradient centrifugation (Lymphoprep) [21]. The purified cells consisted of a more than 95% pure population of viable neutrophils, as assessed by morphology and trypan blue exclusion test.

Neutrophil chemotaxis was evaluated using a modified Boyden chamber assay, with blind well chambers and 3 μm micropore filters (Millipore, Bedford, MA, USA) [22]. Briefly, 200 μl of the cell suspension, containing 2.5 × 10^6 ^neutrophils/ml in RPMI1640 + 0.4% BSA were layered on top of the filter, and the lower compartment was filled with 200 μl of the chemotactic factor (see below). Following incubation at 37°C for 90 min in a humidified atmosphere with 5% carbon dioxide, the filters were fixed with ethanol and stained with haematoxylin–eosin. The chemotactic response was then determined by evaluating the number of cells × high power field that had migrated through the entire thickness of the filter; triplicate chambers were used in each experiment and five fields were examined in each filter. In all cases the person scoring the assay had no knowledge of the experimental grouping. The chemoattractants were zymosan-activated serum (1 mg/ml for 30 min at 37°C) at a 10% (vol/vol) final dilution in RPMI 1640, and the synthetic peptide FMLP at 10^-8 ^mol/l final concentration.

Phagocytosis was evaluated using C3-coated zymosan (C3Zy) as particles for uptake [23]. C3Zy was prepared incubating zymosan in normal human serum (5 mg/ml) for 30 min at 37°C followed by extensive washing. The neutrophil suspension (200 μl) was incubated with C3Zy (cell to particle ratio, 1:5) for 30 min at 37°C in a shaking water bath. Cytocentrifuge slides of the mixtures were then immediately prepared and stained with May Grunwald–Giemsa. The number of particles ingested per cell (phagocytic index [PI]) were established by direct light microscopy (1000 × magnification) of at least 200 cells. In all cases the person scoring the assay had no knowledge of the experimental grouping.

Lucigenin-amplified chemiluminescence was used to evaluate production of ROS by neutrophils [23]. For the measurement of chemiluminescence, 1 × 10^5 ^neutrophils were mixed in 3 ml polystyrene vials with 5 × 10^-5 ^mol/l lucigenin in a final volume of 700 μl. The vials were placed in the Luminometer 1251 (LKB Wallace, Turku, Finland) in the dark and allowed to equilibrate for 5 min at 37°C with intermittent shaking previously to record the background of the light output in mV. PMA (final concentration 5 ng/ml) was added with an appropriate dispenser (1291; LKB Wallace) and chemiluminescence was recorded continuously. Background counts were subtracted from the values obtained after neutrophil stimulation.

Levels of neutrophil membrane expression of CD69 and CD11b were evaluated as previously reported [23]. Briefly, 2 × 10^5 ^neutrophils were washed in phosphate-buffered saline (PBS) and resuspended with 100 μl PBS containing 0.1% NaN_3_, 10% human AB serum (to prevent nonspecific binding of mAb to Fc receptors) and predetermined saturating concentrations of the anti-CD69 or anti-CD11b mAbs. After incubation for 60 min at 4°C the cells were washed twice with PBS/NaN_3_/0.1% BSA and the pellets were resuspended in 100 μl of the same buffer containing FITC-conjugated goat anti-mouse IgG in a saturating concentration and incubated for 30 min at 4°C. The cells were then washed twice in PBS and resuspended in 0.5 ml of ice-cold 2% paraformaldehyde in PBS (pH 7.2). The percentage of neutrophils positive for CD69 or CD11b was quantified within 24 hours on a FACSscan flow cytometer (Becton Dickinson). A relative measure of antigen expression was obtained using the mean fluorescence intensity (MFI), converted from log to linear scale, after subtraction of the cells' autofluorescence and the fluorescence of cells incubated with irrelevant isotype control mAbs.

### Statistical analysis

Data are expressed as mean ± standard error of the mean. Statistical analysis was performed using the Student's t-test for unpaired or paired data as appropriate. *P *< 0.05 was considered statistically significant.

## Results

**Figure 1:**
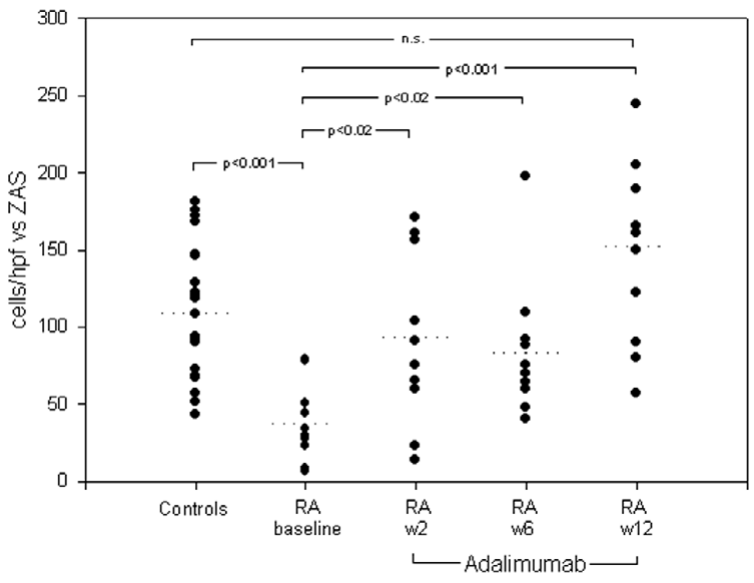
Effect of adalimumab on neutrophil chemotaxis. Peripheral blood neutrophils were purified from 20 controls and 10 patients with rheumatoid arthritis (RA) before (baseline) and during therapy with adalimumab at weeks 2 (w2), 6 (w6) and 12 (w12). Values represent the number of cells migrated × high power field (hpf) using zymosan-activated serum (ZAS) as chemoattractant. The dotted lines indicate the mean values.

**Figure 2:**
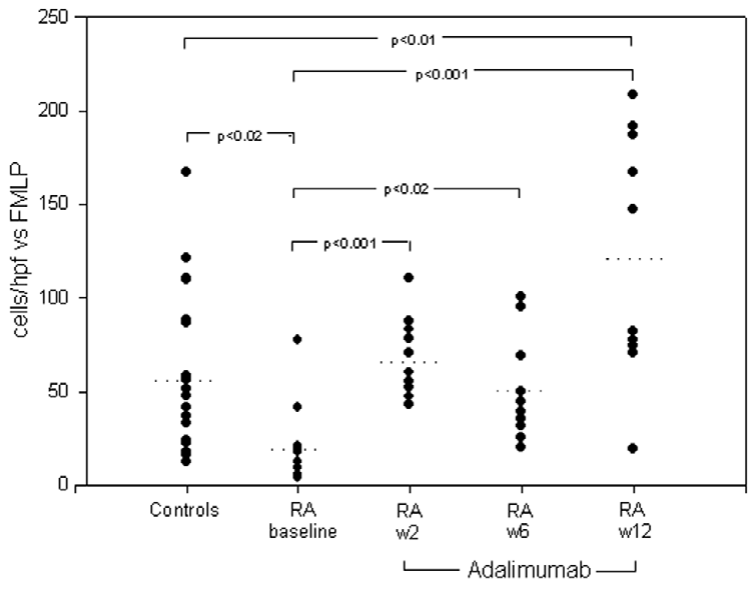
Neutrophils were obtained as described in the legend to Figure 1 and then tested for chemotactic responsiveness toward the chemoattractant *N*-formyl-methionyl-leucyl-phenylalanine (FMLP). The dotted lines indicate the mean values. RA, rheumatoid arthritis.

The chemotactic activity of neutrophils obtained from RA patients at baseline was significantly impaired as compared with that in neutrophils from control individuals; the defect was evident both using zymosan-activated serum (*P *< 0.001; Fig. 1) and FMLP (*P *< 0.02; Fig. 2) as chemoattractant. Two weeks after the start of therapy with adalimumab, the neutrophil chemotactic responsiveness was significantly improved (Figs 1 and 2), with no differences between patients and control individuals. The improvement was evident and persistent during anti-TNF-α therapy at weeks 6 and 12 (Figs 1 and 2).

The phagocytic capacity of neutrophils was similar between control individuals (PI 0.99 ± 0.03) and RA patients at baseline (PI 1.19 ± 0.32), and no changes were observed during anti-TNF-α therapy (week 2: 1.11 ± 0.03; week 6: 1.17 ± 0.09; week 12: 1.03 ± 0.04). The CD11b molecule was spontaneously expressed on more than 90% of neutrophils both in control individuals and in RA patients before and during anti-TNF-α therapy (data not shown). The level of both spontaneous and FMLP-induced CD11b membrane expression (MFI) was also similar between controls (MFI for spontaneous: 155.3 ± 3.7; MFI for FMLP-induced: 591.3 ± 13.9) and RA patients at baseline (MFI for spontaneous: 159.2 ± 8.5; MFI for FMLP-induced: 558.7 ± 27.1), as well as during adalimumab therapy (MFI for spontaneous, week 2: 166.3 ± 12.2; MFI for spontaneous, week 6: 161.0 ± 16.7; MFI for spontaneous, week 12: 154.4 ± 14.9; MFI for FMLP-induced, week 2: 503.6 ± 33.1; MFI for FMLP-induced, week 6: 547.8 ± 27.7; MFI for FMLP-induced, week 12: 610.2 ± 41.8).

**Figure 3:**
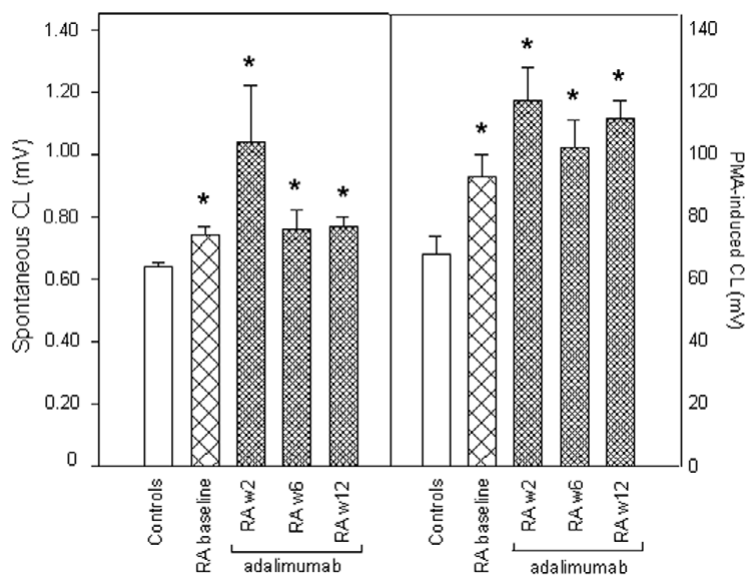
Effect of adalimumab on neutrophil chemiluminescence production. Neutrophils were obtained as described in the legend to Fig. 1 and then tested for chemiluminescence (CL) production in resting conditions (spontaneous CL) or in response to 5 ng/ml phorbol 12-myristate 13-acetate (PMA-induced CL). Results are expressed as mean ± standard error of the mean of peak CL values. *P < 0.05 versus control individuals. RA, rheumatoid arthritis.

Both spontaneous and PMA-induced production of ROS by RA neutrophils was slightly increased at baseline as compared with controls (*P *< 0.05) and the differences persisted at all time points examined during adalimumab therapy (Fig. 3).

**Figure 4:**
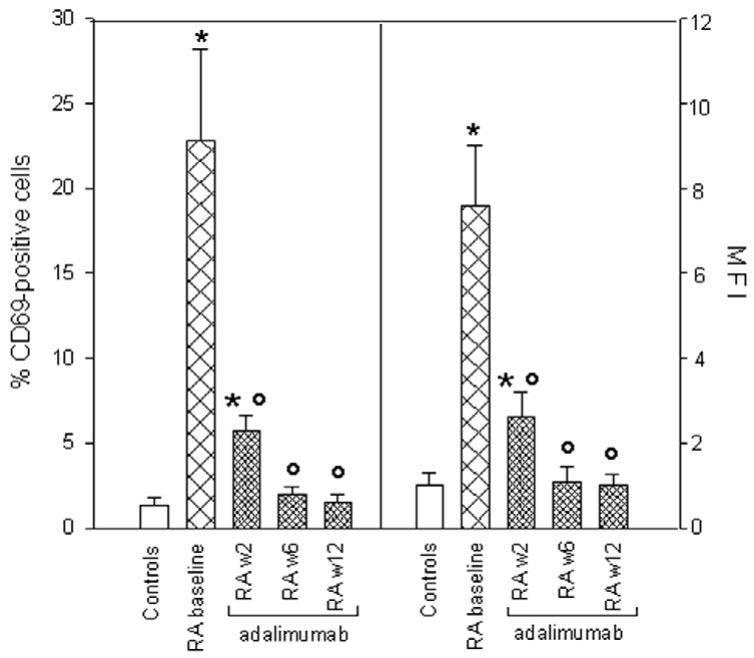
Modulation of CD69 membrane expression on neutrophils by adalimumab. Neutrophils, obtained as described in the legend to Fig. 1, were labelled with anti-CD69 mAb by indirect immunofluorescence. Results are expressed as mean ± standard error of the mean of percentage of positive cells (% CD69-positive cells) and as mean fluorescence intensity (MFI) corrected for nonspecific staining. *P < 0.001 versus controls; °*P *< 0.01 versus rheumatoid arthritis (RA) baseline

Although control neutrophils stained with anti-CD69 mAb yielded very low fluorescence, just above that of unstained cells (%CD69^+ ^cells: 1.3 ± 0.5; MFI: 1.0 ± 0.3), CD69 was significantly expressed on neutrophils from RA patients at baseline (%CD69^+ ^cells: 22.8 ± 5.4; MFI: 7.6 ± 1.4; *P *< 0.001 versus controls; Fig. 4). As shown in Fig. 4, a significant inhibition of CD69 expression on RA neutrophils was induced by adalimumab therapy; the inhibition was already evident at week 2 after the start of therapy (%CD69^+ ^cells: 5.5 ± 0.9; MFI: 2.6 ± 0.6; *P *< 0.01 versus RA baseline) but it was complete at weeks 6 and 12, when no differences were observed between RA patients and control individuals (Fig. 4).

## Discussion

The first aim of the study was to determine whether anti-TNF-α therapy could downregulate neutrophil function, thus reducing the antimicrobial host defence in patients with RA. Our *ex vivo *functional assays do not support this possibility. In fact, we demonstrated that TNF-α neutralization in RA patients did not modify neutrophil activities such as phagocytosis, which were normal at baseline, or ROS production, which was slightly increased at baseline. In agreement with previous studies [24,25], we found impaired chemotaxis of neutrophils from RA patients toward two different chemoattractants. Unexpectedly, TNF-α neutralization induced complete reversal of the neutrophil chemotactic defect. Various mechanisms may account for the defective neutrophil migration in RA patients, such as saturation of membrane receptors with immune complexes [25], cytokine (TNF-α)-induced desensitization [26-28] and drug-induced cell toxicity [29-33]. Of particular relevance are the observations that TNF-α-primed neutrophils are less responsive to chemoattractants [26-28] and are more susceptible to the inhibitory effect of methotrexate on chemotaxis [31]. Because circulating TNF-α has been demonstrated in RA patients [34], it is possible that anti-TNF-α therapy improves neutrophil migration by removing the deleterious effect exerted by soluble and/or membrane bound TNF-α on these cells.

The second aim of the study was to determine whether downregulation of phagocyte activities are involved in the anti-inflammatory activity of anti-TNF-α therapy. The lack of activity on phagocytosis, ROS production or CD11b membrane expression, and the improved migration of neutrophils did not implicate neutrophils as targets of the therapeutic effect of anti-TNF-α. The improved chemotactic responsiveness we observed in patients during adalimumab therapy does not explain the decreased influx of neutrophils into synovial joints previously observed in RA patients during anti-TNF-α therapy [14,15]. However, there is evidence that anti-TNF-α mAbs downregulate the expression of cytokine-inducible adhesion molecules on endothelial cells [35,36]. The decreased activation of endothelial cells in the synovial microvasculature, rather than a defective neutrophil migration, could be responsible for the decreased homing of neutrophils to the inflamed joints.

We recently found that both synovial fluid and peripheral blood neutrophils from RA patients have increased membrane expression of CD69 [37], and this observation was confirmed in the present study. This activation molecule is not constitutively expressed on neutrophils but it may be induced on these cells *in vitro *by several cytokines, such as granulocyte–macrophage colony-stimulating factor, interferon-γ and interferon-α [23,38]. Although a specific ligand for this molecule has not been identified, a role for CD69 in the pathogenesis of RA was previously suggested by Laffon and coworkers [39], who found that CD69^+ ^T lymphocytes were detectable at high levels in synovial fluid and synovial membrane from RA patients and correlated with disease activity. Furthermore, Murata and coworkers [40] recently reported that CD69-null mice were protected from collagen-induced arthritis, and that transfer of neutrophils from wild-type mice could restore arthritis in these animals. These data suggested a crucial role for CD69^+ ^neutrophils in the pathogenesis of arthritis and implicate the molecule as a possible therapeutic target for human arthritis. In the present study we observed that CD69 was downregulated (or inhibited) on neutrophils from RA patients during adalimumab therapy. The mechanism underlying this inhibition is not clear because, in our experience, TNF-α *per se *is not an inducer of CD69 on neutrophils. However, it is possible that other and as yet undefined CD69 inducers are indirectly inhibited by TNF-α neutralization. In agreement with our data, Moore and coworkers [41] recently reported decreased CD69 expression on natural killer cells obtained from mice treated with anti-TNF-α.

## Conclusion

In this study we found that administration of the anti-TNF-α mAb adalimumab to patients with RA does not interfere with the neutrophil activities that are required to maintain an adequate antimicrobial host defence capacity. On the other hand, the inhibitory activity of the mAb on CD69 membrane expression on neutrophils indicates that these cells are among the possible targets of anti-TNF-α activity in RA, and may provide an insight into a new and interesting mechanism of action of anti-TNF-α mAbs in the control of inflammatory arthritis.

## Abbreviations

BSA = bovine serum albumin; C3Zy = C3-coated zymosan; FMLP = *N*-formyl-methionyl-leucyl-phenylalanine; mAb = monoclonal antibody; MFI = mean fluorescence intensity; PBS = phosphate buffered saline; PI = phagocytic index; PMA = phorbol 12-myristate 13-acetate; RA = rheumatoid arthritis; ROS = reactive oxygen species; TNF = tumour necrosis factor.

## Competing interests

The author(s) declare that they have no competing interests.

## Authors' contributions

FC conceived the study, participated in conducting neutrophil functional assays and drafted the manuscript. FM conducted the neutrophil functional assays. PB conducted the immunofluorescence assays. PS-P participated in study design and coordination, and helped to select patients. FA helped with monitoring patients before and during the study. MC participated in coordination of the study. All authors read and approved the final manuscript. AD helped to perform statistical analysis.
